# In vitro ovicidal effect of a *Senecio brasiliensis* extract and its fractions on *Haemonchus contortus*

**DOI:** 10.1186/s12917-019-1843-7

**Published:** 2019-03-25

**Authors:** Suelen Mendonça Soares, Robert Domingues, Emanuelle Baldo Gaspar, Patrício Azevedo dos Santos, Kirley Marques Canuto, Alessandro Pelegrine Minho, Maria Isabel Botelho Vieira

**Affiliations:** 1 Programa de Pós-Graduação em Bioexperimentação, Universidade de Passo Fundo, Passo Fundo, Rio Grande do Sul Brasil; 2 Embrapa Pecuária Sul, Bagé, Rio Grande do Sul Brasil; 3 Curso de Medicina Veterinária, Universidade da Região da Campanha – URCAMP, Bagé, Rio Grande do Sul Brasil; 4 Embrapa Agroindústria Tropical, Fortaleza, Ceará Brasil; 5 Embrapa Pecuária Sudeste, São Carlos, São Paulo Brasil

**Keywords:** Anthelmintic, Egg hatching, Infective larvae migration, Plant extract, Pyrrolizidine alkaloids, Small ruminants

## Abstract

**Background:**

Haemonchosis affects sheep husbandry and its treatment is often compromised due to the development of anthelminthic resistance. Plant-derived bioactive compounds have been studied as alternative to control *Haemonchus contortus*. The objective of this study was to evaluate the effect of *Senecio brasiliensis* extracts on *H. contortus* egg hatching and infective larvae migration.

**Results:**

The aqueous extract from dried and fresh plant and alkaloid-enriched fraction of the previously dried leaves of *S. brasiliensis* inhibited *H. contortus* egg hatching. The main plant compound in alkaloid fraction was integerrimine, a pyrrolizidine alkaloid (PA). However, the aqueous extract from dried plant displayed higher efficacy when compared to their alkaloid enriched or non-polar fractions, meaning that, although PAs contributed to the ovicidal effect, other compounds in the plant can also contribute to their effect. Furthermore, the aqueous extract from dried plant also had higher efficacy than aqueous extract from fresh plant in larvae migration inhibition. Finally, extract from dried plant presented low in vitro cytotoxic effect.

**Conclusion:**

Taken together our results suggest a good anthelmintic effect of *S. brasiliensis*, especially when aqueous extract is prepared from dried plant. Further in vivo studies should be performed focused on forms of administration of this extract in rearing sheep.

## Background

Endoparasitic diseases are a high concern in sustainable sheep production [1]. *Haemonchus contortus* [2] (barber’s pole worm), a blood-sucking nematode and member of the order Strongylida and family Trichostrongylidae, can cause anemia, anorexia, diarrhea, gastritis, and even death in animals [3]. This parasitic infection results in direct economic losses related to decreased animal performance and/or death as well as indirect economic losses linked to the high cost of anthelmintic drugs and the labor and equipment required for the control of parasitosis [4, 5].

*H. contortus* control is mainly based on the use of commercial anthelmintics; however, these drugs have lost their effectiveness due to the development of drug-resistant parasite strains [6, 7]. To aid the control of nematodes, in conjunction with the use of anthelmintics, alternative strategies have been developed, such as pasture management, nutritional adjustments, genetic selection, use of nematophagous fungi, and the development of plant-derived anthelmintic compounds [8, 9].

The genus *Senecio* (Asteraceae) comprises approximately 1500 species [10]. Plants belonging to this genus are known for the production of compounds such as alkaloids, sesquiterpenes, and flavonoids [11] and for their anti-inflammatory, vasodilator, antiemetic, antimicrobial, and parasiticide activities [12–14].

*Senecio brasiliensis* is a native species found in south and southeast of Brazil [15]. It is toxic to the livestock [16, 17] due to the presence of pyrrolizidine alkaloids (PAs), which are widely found in the Asteraceae, Boraginaceae, and Fabaceae families [18, 19]. However, the leaves and inflorescence of *S. brasiliensis* are used in traditional medicine for the treatment of stomach pain [20], a practice justified by the antiulcerogenic and cytoprotective effect of PAs [21, 22].

Based on reports of the pharmacological effects of the genus *Senecio* and the species *S. brasiliensis*, we aimed to evaluate the effect of aqueous extract of this plant and its non-polar and alkaloid fractions on *H. contortus* egg hatching and infective larvae migration, besides evaluating the cytotoxic effect of this plant.

## Results

The GC-MS analysis showed the presence of four constituents for the alkaloid fraction (AF) from dried plant (DP) and eight constituents for AF from fresh plant (FP) (Table 1). In AF from DP, all peaks were attributed to PAs. On the other hand, in AF from FP, in addition to the alkaloids, carboxylic acids and ketones were found. For both extracts, the major compound was integerrimine (~ 91% in AF from DP and ~ 79% in AF from FP).

**Table 1:** GC/MS analysis of *Senecio brasiliensis* alkaloid fraction from dried and fresh plant

Peak	tR	IK	Name of the compound	Peak area (%)
Alkaloid Fraction from Dried Plant				
1	17.020	1464	Senecionine	3.34
2	18.114	1436	Integerrimine	91.14
3	18.400	1456	Platyphylline	1.46
4	19.704	1444	3(2H)-isoquinolinone, octahydro-, (4ar-trans)	4.06
Alkaloid Fraction from Fresh plant				
1	4.830	1381	Cyclohexanone, 5-methyl-2-(1-methylethylidene)	4.15
2	5.721	1491	4-amino-2-nitro-benzaldehyde oxime	0.84
3	7.178	1648	1,2-cyclopropanedicarboxylic acid, 3-(1-methylethenyl)-, diethyl ester	4.12
4	8.959	1406	2-(4-nitrobutyryl) cyclo-octanone	1.19
5	17.071	1363	Senecionine	4.45
6	18.254	1446	Integerrimine	79.26
7	19.068	1500	2-anilino-4-methylquinoline	0.82
8	19.73	1445	Neo-triangularine	1.5

*GC/MS* Gas Chromatography-Mass Spectrometry, *tR* retention time, *IK* Kovats Index

**Fig. 1:**
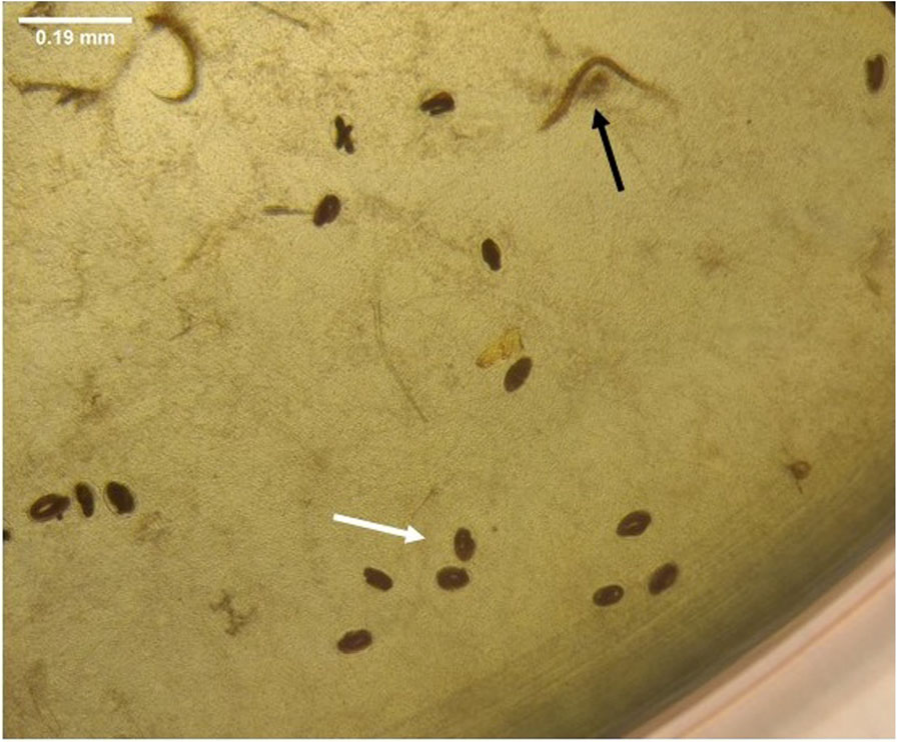
Optical microscopy of eggs and hatched larvae: representative image of *H. contortus* eggs with larva failing eclosion (white arrow) and larva (black arrow). Eggs were treated with dried plant extract during 24 h. 100x magnification

**Fig. 2:**
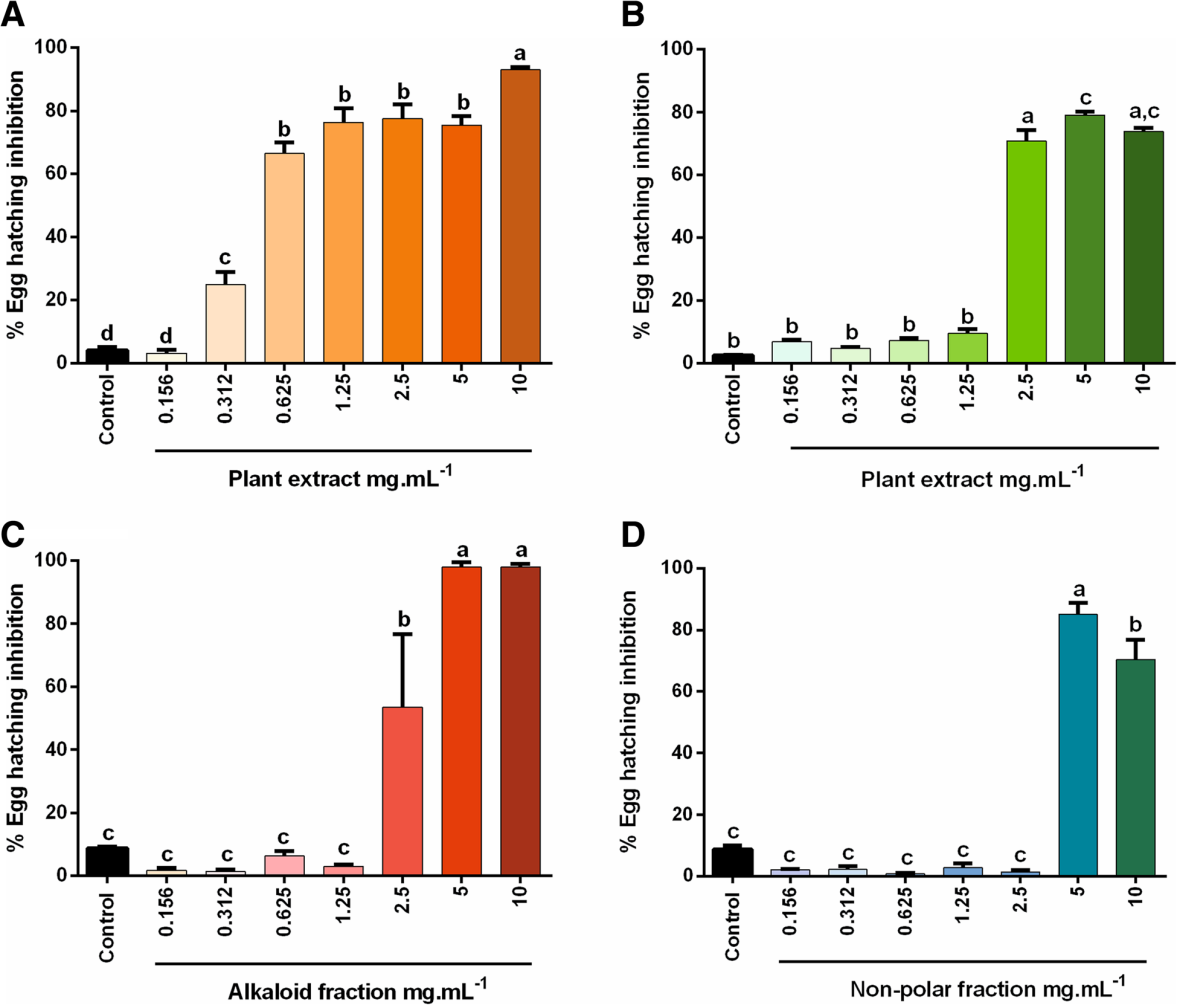
Effect of *Senecio brasiliensis* extracts on *Haemonchus contortus* egg hatching inhibition: **a** Dried plant extract (DP); **b** Fresh plant extract; **c** Alkaloid fraction of DP extract; **d** Non-polar fraction of DP extract. Different letters mean difference between groups (*p* < 0.05; one-way ANOVA followed by Tukey’s post-hoc test)

The effective concentrations to inhibit *H. contortus* egg hatching (Fig. 1) by 50% (EC_50_) were 0.660 mg.mL^− 1^ for DP and 2.596 mg.mL^− 1^ for FP, respectively (Table 2). DP showed high inhibitory activity at the highest concentration tested (94%) (Fig. 2a), whereas FP showed moderate inhibitory activity at the highest concentration tested (73%) (Fig. 2b). The percentage of inhibition of egg hatching ranged from 1 to 94% for DP and from 4 to 73% for FP. For DP aqueous extract, inhibition rates above 50% were observed for concentrations higher than 0.625 mg.mL^− 1^ (Fig. 2a). On the other hand, for FP, significant egg hatching inhibition was observed only for concentrations above 2.5 mg.mL^− 1^ (Fig. 2b).

**Table 2:** Mean (SE) of effective concentration to inhibit 50% (EC_50_) of egg hatching or larval migration and to kill 50% of the cells

Extract	In vitro test^b^	EC_50_ (mg.mL^−1^)
Dried plant	EHT	0.660 (0.506 to 0.859)
	LMIT	10.150 (8.022 to 12.840)
	CT	45.430 (37.580 to 54.920)
Alkaloids from dried plant	EHT	2.435 (2.143 to 2.765)
Non-polar fraction from dried plant	EHT	4.356 (0.434 to 43.760)
Fresh plant	EHT	2.596 (2.139 to 3.086)
	LMIT	1712 (44.020 to 66.560)
Alkaloids from fresh plant	EHT	^a^
Non-polar fraction from fresh plant	EHT	^a^

^a^It was not possible to estimate

^b^*EHT* Egg Hatching Test, *LMIT* Larval Migration Inhibition Test, *CT* Cytotoxicity Test

The estimated EC_50_ was 2.435 mg.mL^− 1^ for the AF from DP. Non-polar enriched fraction (NPF) from DP showed EC_50_ of 4.356 mg.mL^− 1^. The EC_50_ values for AF and NPF from FP could not be determined, due to low effect, since the eclosion inhibition was less than 10% in all tested concentrations. At the highest evaluated concentrations, AF (Fig. 2c) and NPF (Fig. 2d) from DP exhibited egg hatching inhibition of 98 and 70%, respectively. The average inhibition for AF from DP ranged from 1.7 to 98% and started at 2.5 mg.mL^− 1^, while the average inhibition for NPF from DP ranged from 0.5 to 70% and started at 5 mg.mL^− 1^. The egg hatching inhibition in the positive control (thiabendazole 5 mg.mL^− 1^) was 98%.

**Fig. 3:**
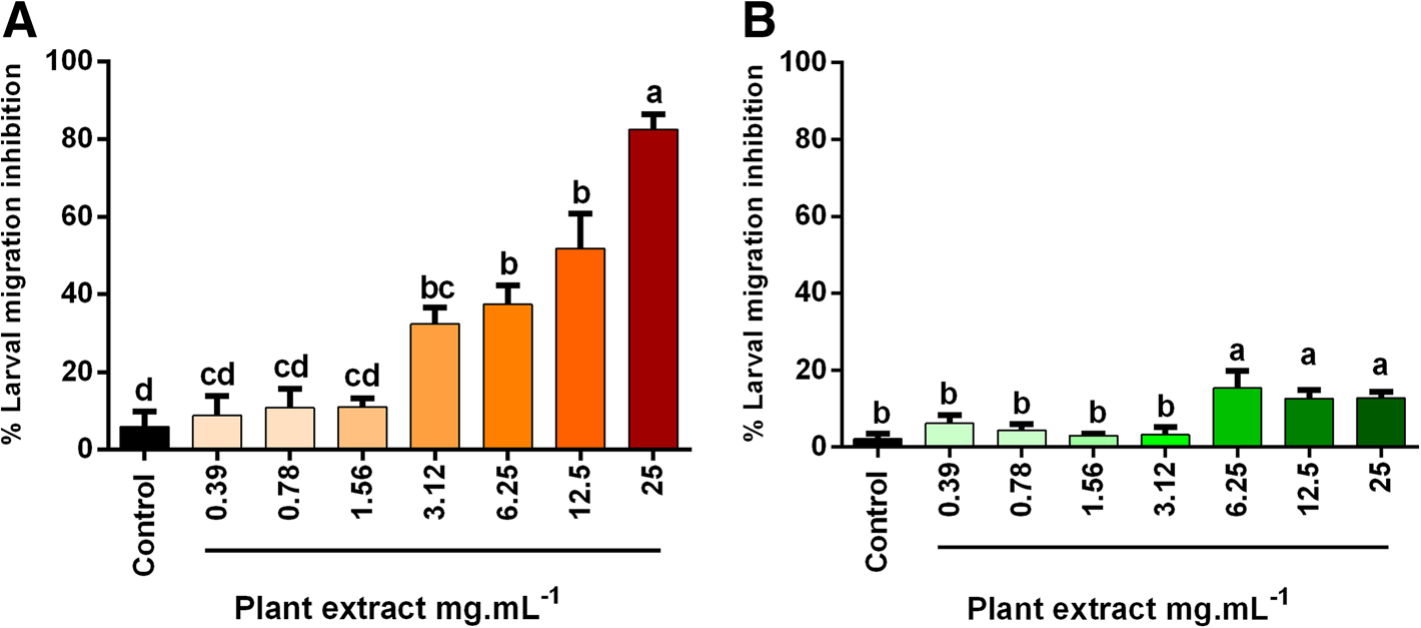
Effect of *Senecio brasiliensis* extracts on *Haemonchus contortus* larval migration inhibition: **a** Dried plant extract; **b** Fresh plant extract. Different letters mean difference between groups (*p* < 0.05; one-way ANOVA followed by Tukey’s post-hoc test)

Concerning larval migration inhibition, the EC_50_ were 10.15 mg.mL^− 1^ for DP and 1712 mg.mL^− 1^ for FP (Table 2). The maximal larval migration inhibition, observed when high concentrations of plant extract were used, was 15% for FP and 82% for DP. The percentage of inhibition migration ranged from 6 to 82% for DP and from 2 to 15% for FP. For DP, migration inhibition rates above 50% were observed only in concentrations higher than 12.5 mg.mL^− 1^ (Fig. 3a). On the other hand, no migration inhibition above 50% for FP was observed (Fig. 3b). The larval migration inhibition of the positive control (levamisole 6.25 μg.mL^− 1^) was 96%.

**Fig. 4:**
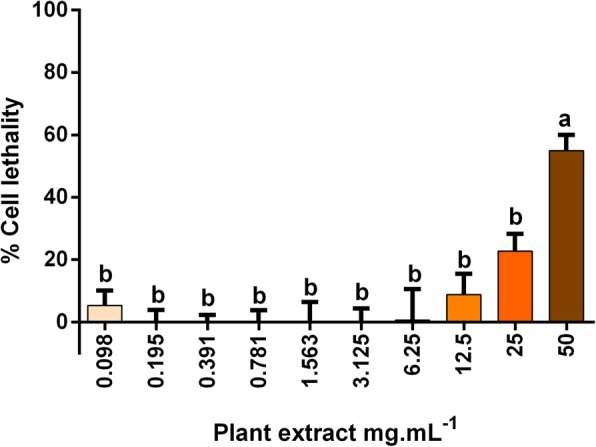
Cytotoxic effect of dried *Senecio brasiliensis* extract on Vero cells. Different letters mean difference between groups (*p* < 0.05; one-way ANOVA followed by Tukey’s post-hoc test)

A dose-dependent pattern is observed for cytotoxic activity of DP aqueous extract (Fig. 4). However, the effect of this extract in mammal cell lethality was pronouncedly less intense than for egg hatching or larval migration inhibition, as demonstrated by the SI that was 68.63 and 4.48, respectively. The cytotoxic EC_50_ of DP was estimated in 45.43 mg.mL^− 1^ (Table 2). Maximal cell lethality was 54.94% when 50 mg.mL^− 1^ of DP extract was used.

## Discussion

Integerrimine, found in plant extracts, is among the main PAs in *Senecio* [22–24]. Although integerrimine was the most abundant component in both AFs, its concentration was higher in AF from DP as compared to AF from FP, possibly because the drying procedure at 40 °C increased the concentration of some compounds while caused the loss of others, what could also be a plausible explanation for the reduction of compounds diversity in AF from DP when compared to AF from FP. Previous studies have shown that hot-drying can affect the chemical composition of plant extracts when compared with those obtained by freeze-drying (lyophilization) [25, 26].

An anthelmintic is considered effective if it exhibits at least 90% of inhibitory activity [27]. Considering this cut-off, DP and AF from DP from *S. brasiliensis* showed efficacy against egg hatching of *H. contortus* isolated from sheep feces. In our experiment we observed the phenomenon ‘failing eclosion’, described by Vargas-Magaña et al., [28] and Chan-Pérez et al., [29] in studies with plant extracts. These authors used this term to differentiate larval eggs from morulated eggs, which were classified as dead. In our work we observed the presence of morulated eggs only in the test with the positive ovicidal control Thiabendazole. We believe that the extract of *S. brasiliensis* prevents the larva exit of the egg, by mechanisms still unknown. Regarding larval migration inhibition, it is not possible to conclude an effectiveness of *S. brasiliensis* extracts, since at the tested concentrations; the highest inhibitory activity observed was 82.6%, observed for DP aqueous extract. Taking into consideration the effect of DP on egg hatchability, we believe that, in further studies, *S. brasiliensis* extract should be considered to be used in reduction of pastures contamination, acting as an environmental control, as observed by Niezen et al., [30].

Among the set of alkaloids identified in AF from DP, PAs were the most abundant, suggesting their participation in the observed effect. In addition, the efficacy of those substances in combating plant nematodes has already been reported [31]. Surprisingly, the effect of DP (EC_50_ = 0.660 mg.mL^− 1^) was higher than AF from DP (EC_50_ = 2.435 mg.mL^− 1^), suggesting that PAs are not the only responsible components for anthelmintic effects observed, which has already been reported for other plant extracts. In the study conducted by Brandão et al. [32], the total extract of *Bidens* sp. (Asteraceae) was more effective in reducing *Plasmodium* sp. parasitemia than its fractions. Gomes et al. [33] also recorded a similar event in their in vitro study with *Zizyphus joazeiro* against *H. contortus*; the saponins found in a plant fraction acted together with other substances present in the total extract. Saponins, tannins, phlobatannins, phenols, anthraquinones, flavonoids, glycosides, steroids, terpenes, cardenolides, and chalcones [34, 35] have been identified in other *Senecio* species and some of these compounds have been associated with anthelmintic effects [36–38].

Even according to the traditional medicine, whole plants are used rather than isolated compounds. This phenomenon can be attributed to the synergic effect, what occurs when the combination of components of a plant extract or mixture of plant extracts effect is greater than the sum of individual effects [39]. Although synergy is a well-documented effect, there is a lack in acknowledgment concerning its mechanism of action [40]. An example of synergy can be found in the work of Klongsiriwet et al., [41], who detected a greater inhibition of the exsheathment of *H. contortus* infective larvae when tannins and flavonoids were mixed, in relation to the effect of these compounds in the isolated form. However, these authors only speculated how the synergy occurred, as Williamson [40] and Wagner and Ulrich Merzenich [42] cite in their works. Although we have not performed experiments with isolated components and their possible mixtures, we suppose that DP is more potent than its fractions due to some degree of synergy between the alkaloids and the other components present in aqueous extract of the plant.

Despite *S. brasiliensis* having ovicidal effect, this plant may be toxic to animals [43], which may be attributed to liver metabolism of pyrrolizidine alkaloids. Three metabolic ways may occur with PA in liver: dehydrogenation, hydrolysis and n-oxidation [44, 45]. According to these authors, dehydrogenation forms pyrroles that are toxic, whereas hydrolysis and n-oxidation leads to non-toxic metabolites. The pyrroles originating from dehydrogenation of PAs can bind with the hepatocyte DNA, inhibiting cellular mitosis and thereby causing hepatic dysfunction [46, 47]. However, it is known that sheep, the targets of our study, are more resistant to *Senecio* intoxication than cattle due to their ruminal microbiota [48] and liver metabolization system [49].

It worth mentioning that in vitro anthelmintic and cytotoxic effects of *S. brasiliensis* were conducted with plant extracts in a non-metabolized form. The hypothesis of animal toxicity after liver metabolization of the compounds corroborate our results, in which in vitro cytotoxicity was low [50, 51] and which confirms previous researches that claim that alkaloids are not toxic in the non-metabolized form [44, 52]. Thus, future studies involving new formulations in order to avoid liver metabolization or to protect that compounds against organism degradation need to be carried out.

## Conclusions

DP of *S. brasiliensis* displays good anti-*H. contortus* egg hatchability and, less pronouncedly, anti-larvae activity, presenting low in vitro cytotoxicity to Vero cells. Future studies are required to clarify the mechanism of action on *H. contortus* eggs and the pharmacological mechanisms of the plant extract in animals to validate the DP uses in integrated nematodes control programs.

## Methods

### Reagents

Sodium hydroxide (NaOH), sulfuric acid (H_2_SO_4_), dichloromethane (CH_2_Cl_2_), sodium sulfate (Na_2_SO_4_), and methanol (MeOH) (High Performance Liquid Chromatography grade) were purchased from Vetec® (Brazil). For the parasitological tests, thiabendazole, tween® 80, levamisole, sodium hypoclorite (NaOCl) and sodium chloride (NaCl) were purchased from Sigma-Aldrich (USA). Rezasurin used in cytotoxicity evaluation was also purchased from Sigma-Aldrich (USA).

### Plant material

*S. brasiliensis* was collected from fields that belong to Embrapa Pecuária Sul, located in the city of Bagé, Rio Grande do Sul, Brazil (31°21′13.3″S 54°00′36.2″W). A voucher specimen (103517) was stored in the herbarium of the Embrapa, Brasília, Distrito Federal, Brazil.

### Extraction and fractionation

#### Aqueous extract from fresh plant (FP)

Leaves of *S. brasiliensis* were collected, immediately macerated and mixed with water (100 mg.mL^− 1^) at room temperature. The material was filtered through cotton, concentrated by freeze-drying using a lyophilization apparatus (LP510, Liotop®, Brazil), and stored at − 20 °C.

#### Aqueous extract from dried plant (DP)

Collected leaves of *S. brasiliensis* were dried at 40 °C for 2 days. Subsequently, the material was macerated, powdered, and mixed with distilled water (40 °C for 30 min) to make a 100 mg.mL^− 1^ stock solution. The solution was filtered through cotton, concentrated by lyophilization, and stored at − 20 °C.

#### Alkaloid and non-polar fractions

The alkaloid and non-polar organic fractions (AF and NPF, respectively) were prepared according to the procedure described by Torras-Claveria et al. [53] and Andrade et al. [54] with some modifications. Lyophilized samples were dissolved in 40 mL of MeOH by sonication (Sanders, Brazil). Next, the sample was acidified to pH 2 with 2% H_2_SO_4_, and the organic fraction was removed by mixing with CH_2_Cl_2_ followed by decantation. This procedure was repeated three times. The lower fraction was enriched with non-polar compounds and was called NPF. The upper fraction (polar) was basified up to pH 11 with 10% NaOH, and the alkaloids were extracted by liquid-liquid partition with CH_2_Cl_2_ three times. Afterwards, the lower fractions were enriched with alkaloids compounds and called AF. Finally, the NPF and AF were concentrated in a rotary evaporator (Tecnal, Brazil).

#### GC-MS analysis of alkaloid extracts

The dried alkaloid-enriched extracts were dissolved in CHCl_3_ and directly injected into the GC-MS (Gas Chromatography-Mass Spectrometry) apparatus (injection volume: 1 μL) consisting of a Hewlett Packard 6890 coupled with a mass spectroscopy device (5975 GC/MS, Hewlett Packard, USA) operating in EI (Ionization by Electron) mode at 70 eV. An HP-5 MS column (30 m × 0.25 mm i.d., film thickness 0.25 m) was used. The temperature gradients were as follows: 100–180 °C at 15 °C/min, 180–300 °C at 5 °C/min, 10 min hold at 300 °C and 2 min at 100 °C. The injection temperature was 250 °C, and the flow rate of the carrier gas (helium) was 1 μL/min. A split ratio of 1:5 was applied. Furthermore, the arithmetic retention indexes were calculated by linear interpolation relative to the retention times (tR) of a series of n-alkanes (C7–C30). The obtained values were compared with the published retention index (Kovats index) values [55, 56]. Mass spectra were deconvoluted using AMDIS® 2.64 software (NIST) [54].

### Parasitological tests

#### Animals

Eggs were obtained from the feces of sheep infected and maintained with a monospecific culture of *H. contortus*. These animals belong to the Embrapa Pecuária Sul, which has own sheep farming, and where the research was carried out. The recovery of eggs was performed according to the method described by Coles et al. [57]. Feces were homogenized in water and filtered through sieves of diminishing pore diameter (180, 90, 68, 38 μm), and the eggs were separated by centrifugation (3000 rpm for 5 min) in a saturated NaCl solution. Immediately, the eggs were washed with distilled water and used in experiments. The experimental protocol was in accordance with the directives of Brazilian National Experiment Control Council (Ethics Committee on Use of Animals – Embrapa Pecuária Sul, protocol under registration number 01/2017). To obtain third stage larvae, the eggs obtained in the same manner as described above were incubated under aerobic conditions in fecal cultures for 7 days at 28 °C and 80–85% humidity. Larvae obtained from these cultures were isolated using a Baermann funnel system and larval cultures were stored at 4–8 °C. After experiments, the animals were treated with anthelmintics and returned to the Embrapa Pecuária Sul fields.

#### Egg hatching test

The in vitro egg hatching test (EHT) was based on the method described by Coles et al. [58] and standardized by von Samson-Himmelstjerna et al. [59] with modifications. DP and FP were diluted in distilled water and AF and NPF were diluted in 5% tween® 80 aqueous solution. Diluents alone were used as negative controls. Thiabendazole (5 mg.mL^− 1^) was used as positive control, diluted in 5% tween® 80. Approximately 120 *H. contortus* eggs diluted in water or 5% tween® 80 were incubated with final concentration of 10, 5, 2.5; 1.25, 0.625, 0.312, and 0.156 mg.mL^− 1^ of DP, FP, AF or NPF in a BOD incubator, for 24 h at 28 °C and 80% humidity. Next, 1% lugol’s iodine was added to each well, and the eggs and larvae at the first stage (L1) were microscopically quantified. The test was only validated when hatching in the negative control was greater than 90%. To calculate the percentage of inhibition of larval hatching [58], the formula [(A)/(A + B)] × 100 was used, where A = number of eggs and B = number of larvae. The data were corrected using the Abbott formula [60]. Tests were performed in quadruplicate.

#### Larval migration inhibition test

Larval migration inhibition test (LMIT) was performed according to Demeler et al., [61, 62] with some modifications. DP and FP were diluted in distilled water, which alone was used as negative control. Levamisole (6.25 μg.mL^− 1^) was used as positive control, diluted in distilled water. About 120 third stage larvae, previously exsheathed with a solution of sodium hypochlorite (0.3%), were incubated in seven different concentrations of plant extract (25; 12.5; 6.25; 3.12; 1.56; 0.78 and 0.39 mg.mL^− 1^) in 24 well plates in a final volume of 1000 μL per well. After 24 h at 28 °C, the total volume (extract + larvae) was deposed into and 25-μm sieves mounted on wells and incubated for more 24 h at 28 °C. After the second incubation, sieves were carefully removed and the remaining non-migrated larvae were washed with distilled water into another well. 1% lugol’s iodine was added to each well and migrated and non-migrated larvae were quantified at 100x magnification. The percentage of non-migrated larvae to the total amount of larvae was calculated for the controls and every concentration tested, as mentioned in EHT, with A = number of non-migrated larvae and B = number of migrated larvae and Abbott formula [60] also was used. Tests were performed in quadruplicate.

#### Cell citotoxicity assay

Vero cells were maintained in RPMI media (Cultilab, Brazil) supplemented with penicillin (100 UI.mL^− 1^) (Sigma-Aldrich), streptomycin (0.01 mg.mL^− 1^) (Sigma-Aldrich), gentamycin (10 μg.mL-1 (Sigma-Aldrich), and 10% bovine calf serum (Cultilab). Confluent cells were trypsinized and seed on 96 well plates, totalizing 1.5 × 10^4^ cells/well. Four hours later, when cells were completely adhered, test solutions were added. Two fold serial dilutions were carried out with DP diluted in distillated water to obtain final concentrations ranging from 50 to 0.098 mg.mL^− 1^. The microplates were sealed and incubated at 37 °C in normal atmosphere during 24 h. After this period, resazurin solution (Sigma-Aldrich) was added to each well, to a final concentration of 0.3 mM and the plates were incubated at 37 °C for additional 18 h. The absorbance values were read by dual wavelength using a microplate spectrophotometer (Versa-Max, Molecular Devices) at 570 and 600 nm. All experiments were performed three times each in triplicate. Percentage of viable cells (VC) was calculated as described by Rolón et al., [63]. Percentage of citotoxicity was calculated as 100-VC and was used to calculate EC_50_. Selectivity index (SI), the ratio between cytotoxicity EC_50_ and egg hatching or larval migration inhibition EC_50_, was calculated to evaluate the safety of the extract tested [50].

#### Statistical analysis

An analysis of variance (ANOVA) followed by Tukey’s test was performed on the test results. A *p-*value ≤0.05 indicated statistical significance. A logistic equation with a variable slope was used to fit the dose-response data by non-linear regression and find the EC_50_. All analyses were performed after transforming the concentrations into their logarithms (X = log X) and constraining the bottom value to 0% and top value to 100%. All statistical analyses were performed using GraphPrism® version 6.07 (GraphPad Software, USA).

## Abbreviations

°C: Celsius degree
AF: Alkaloid fraction
DNA: Deoxyribonucleic acid
DP: Dry plant
EC: Effective concentration
EHT: Egg hatching test
FP: Fresh plant
GC-MS: Gas Chromatography-Mass Spectrometry
*H. contortus*: *Haemonchus contortus*
LMIT: Larval migration inhibition test
mg.mL^−1^: Milligram per mL
mM: Millimolar
NaCl: Sodium chloride
nm: Nanometers
NPF: Non-polar fraction
PA: Pyrrolizidine alkaloid
*S. brasiliensis*: *Senecio brasiliensis*
SI: Selectivity index
